# An automatic system for crystal growth studies at constant supersaturation

**DOI:** 10.1155/S1463924692000336

**Published:** 1992

**Authors:** J. G. March, A. Costa-Bauzá, F. Grases, O. Söhnel

**Affiliations:** 1 Department of Chemistry University of the Balearic Islands Palma de Mallorca 07071 Spain; 2 Department of Inorganic Processes Institute of Technology Pardubice 532 10 Czech Republic

## Abstract

An automatic system for growing crystals from seeded supersaturated solutions at constant supersaturation is described. Control of burettes and data acquisition are controlled by computer. The system was tested with a study of the calcium oxalate kinetics of crystal growth.


Journal of Automatic Chemistry, Vol. 14, No. 5 (September–October 1992), pp. 177–180

### An automatic system for crystal growth studies at constant supersaturation

**J. G. March, A. Costa-Bauzá, F. Grases**

*Department of Chemistry, University of the Balearic Islands, 07071 Palma de Mallorca, Spain*

**and O. Söhnel**

*Department of Inorganic Processes, Institute of Technology, 532 10 Pardubice, Czechoslovakia*

*An automatic system for growing crystals from seeded supersaturated solutions at constant supersaturation is described. Control of burettes and data acquisition are controlled by computer. The system was tested with a study of the calcium oxalate kinetics of crystal growth.*

### Introduction

Understanding the mechanism of crystal growth has important implications for industry [1,2] and in the appreciation of biomineralization processes [3]. Studies on the mechanism of crystal growth generally use seed crystals; growth rates are monitored either by following the decrease in lattice ion concentration (decreasing supersaturation conditions), or by creating constant supersaturation using continuous crystallizers [4,5]. Supersaturation appears to govern crystal growth so experiments in which crystals grow at constant supersaturation are particularly interesting.

An automatic system for crystal growth studies, where the range of oscillation of the supersaturation during the crystallization process can be automatically controlled from potentiometric measurements, is presented in this paper. The surface area of crystals was the only variable which did not have a constant value during the experiments. A method of calculating crystal growth rates per unit of crystal area is also explained in the paper.

Calcium oxalate was chosen to test the automated system because its growth has been extensively studied [6,8].

### Experimental

### *Reagents*

Solutions of calcium chloride (Panreac, Barcelona, Spain), ammonium oxalate and ammonium chloride (Probus, Barcelona, Spain), were prepared with reagent-grade chemicals. The calcium oxalate monohydrate used to seed the crystallization cell was purchased from Panreac.

Seed suspensions were prepared with 2 g calcium oxalate monohydrate and 24 ml distilled water.

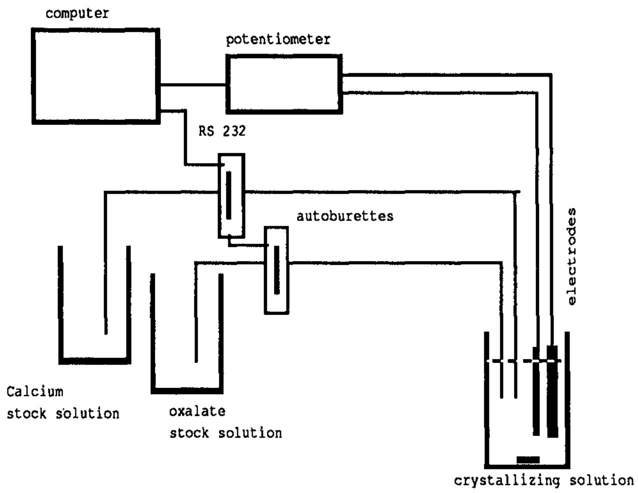

*Figure 1. Schematic diagram of the automatic crystallization system described in the paper.*

### *Apparatus*

The automated crystal growth system (see figure 1) included the following:

(1) A Macintosh SE computer with an RS 232C interface.

(2) A micropotentiometer Crison 2002 with a RS 232 interface and two microBUR autoburettes connected by a daisy-chain system supplied by Crison Instruments (Barcelona, Spain).

(3) A calcium ion selective electrode (Ingold), coupled with a silver/silver chloride electrode separated from the solution by an intermediate junction containing potassium chloride.

(4) A 600 ml reaction glass vessel with four wall baffles and a magnetic stirrer; the vessel was placed in a constant temperature water bath at 37 °C.

### *Crystal growth experiments*

The experimental procedure involved preparing an equimolar metastable supersaturated solution of calcium oxalate ($c_0$ and $V_0$ are used to denote its initial molar concentration and volume). This solution contained ammonium chloride to adjust the ionic strength (I = 0·1M) in order to meet the operating instructions for the calcium ion selective electrode. The solution was kept under stirring at the appropriate temperature until the electrode reading was constant for 10 min. This constant potential value ($mV_{constant}$) was set as the predetermined value and maintained throughout the run.

0142–0453/92 $3.00 © 1992 Taylor & Francis Ltd.

The solution was then seeded by adding 1 ml of a homogeneous suspension of calcium oxalate monohydrate in water. $W_o$ denotes the weight of added solid phase. When the seed crystals started growing, the concentration of calcium and oxalate decreased producing a reduction in the electrode potential below the $mV_{constant}$ value. The automatic system then runs additions of 0·1 ml aliquots of equimolar stock solutions of calcium and oxalate until the predetermined $mV_{constant}$ value is achieved. $c_s$ denotes the molar concentration of stock solutions, and $V_a$ the total volume added to the crystallizing suspension.

Assuming that reactions (ion association and crystallization) take place at 1 : 1 molar relationship, each electric potential value corresponds to only one supersaturation value. Then, the oscillation of the electric signal, which is related to both the oscillation of the supersaturation value and the rate of additions of stock solution aliquots, depends mainly on the stock solution concentration and on the supersaturation corresponding to $mV_{constant}$. It is necessary, therefore, to discover the concentration at which too wide an oscillation of potential and too low a rate of aliquot addition is avoided.

The number of additions and corresponding times were recorded for later computer processing.

Samples of the reaction mixture (5 μl) were occasionally withdrawn and crystals observed by scanning electron microscopy to study the evolution of the solid phase. The crystallization cell was continuously stirred, and the temperature was kept constant.

*System control and data-acquisition*

The software used was Lab View 2 (Logical Balear Co., Palma de Mallorca, Spain). Three functions were controlled in this way: acquisition of mV data; control of autoburettes and acquisition of number of addition and time data. The program flowchart is shown in figure 2. The predetermined mV constant value ($mV_{constant}$) is entered and if:

$$mV < mV_{constant} \quad (1)$$

then the program runs one addition of 0·1 ml from both autoburettes to the crystallization cell. Additions continue until the operator stops the program. The third function of the program is to write to file the additions and accumulated time data; this information can also be printed out.

*Theoretical considerations*

Crystal growth parameters can be calculated with the automatic system. The following explains the theory behind the calculation of crystal growth rates:

(1) Working under appropriate experimental conditions, and for a time interval ($\Delta t$) including several additions, the cation and anion average concentration in the crystallizing suspension can be assumed to be constant and equal to the initial concentration ($c_o$). Mathematically:

$$\frac{n_{initial} - n_{crystallized} + n_{added}}{V_o + V_a} = \frac{n_{initial}}{V_o} \quad (2)$$

where $n$ is the number of moles.

(2) When crystals are growing, the number of microcrystals ($N$) remains constant. Thus the addition of calcium oxalate to the seed crystals leads to an increase in weight ($W$) and surface area ($A$). Assuming that the crystals are spherical shape with an average radius of $r$ the surface area and weight of the crystal phase in suspension can be expressed as:

$$A = k_a N r^2 \quad (3)$$

$$W = k_w N r^3 \quad (4)$$

where $k_a$ and $k_w$ are proportionality constants.

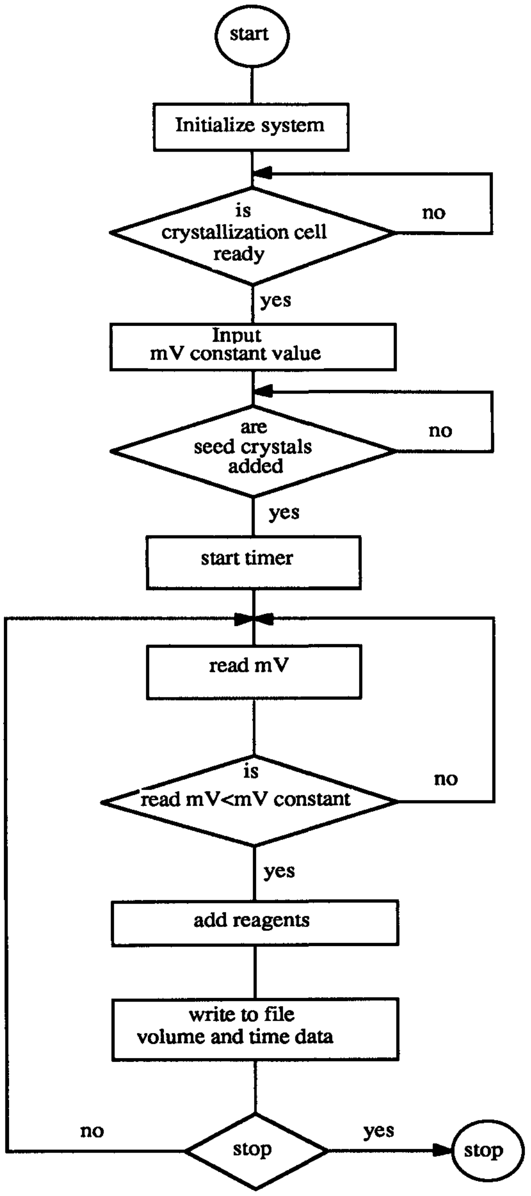

*Figure 2. Computer program used to control supersaturation conditions during the crystallization process.*

(3) The increase in crystal weight taking place in suspension ($\Delta W$) can be calculated from the experimental data. Taking into account the dilution taking place during additions, then:

$$\Delta W = M_w V_a(c_s/2 - c_o) \qquad (5)$$

where $M_w$ is the molecular weight of the precipitating salt.

(4) In constant supersaturation seeded experimental conditions, the rate of crystal growth ($R$) can be taken to be proportional to the calcium (or oxalate) mole number incorporated to the crystal phase ($n_{\text{crystallized}}$) per unit of time ($\Delta t$) and unit of area of crystal phase ($A$):

$$R = k_r \frac{n_{\text{crystallized}}}{\Delta t . A} \qquad (6)$$

where $k_r$ is a proportionality constant.

Combining equations (3), (4), (5) and (6), $R$ can be expressed as:

$$R = \frac{kr}{A_o} \frac{\Delta V(c_s/2 - c_o)}{\Delta t} \left[1 + \frac{M_w V_a(c_s/2 - c_o)}{W_o}\right]^{-2/3} \qquad (7)$$

where $A_o$ is the total area corresponding to the seed crystals which were added to start the process, and $\Delta V$ is the added volume corresponding to $\Delta t$.

If the area of the solid phase is assumed to be constant, equation (7) transforms into

$$R = \frac{k_r}{A_o} \frac{\Delta V(c_s/2 - c_o)}{\Delta t} \qquad (8)$$

Alternatively, crystal growth rates can be adjusted to a power law as:

$$R = k(\text{supersaturation})^g \qquad (9)$$

where $k$ is the apparent kinetic constant and $g$ is defined as the apparent order of the crystal growth process.

When looking at the kinetics of crystal growth, the order of reaction is of great interest because its value is related to the predominant mechanism of crystal growth (surface nucleation/screw dislocation growth). The methodology proposed in this article takes supersaturation as a known experimental variable, so $g$ can be calculated using equation (9).

### Results and discussion

The system described in this paper allows the crystallization of sparingly soluble salts at controlled supersaturation conditions to be studied. Calcium oxalate monohydrate was used to test the system. The system performed satisfactorily for the determination of crystal growth rates, order and formal kinetic constants for crystal growth processes, and for studying kinetic evolutions of the solid phase (for example size and morphology) as a function of the supersaturation. Selected preliminary experiments at different supersaturation conditions are presented in figures 3 and 4. Crystal growth rates were calculated using equations (7) and (8) and the results have been compared.

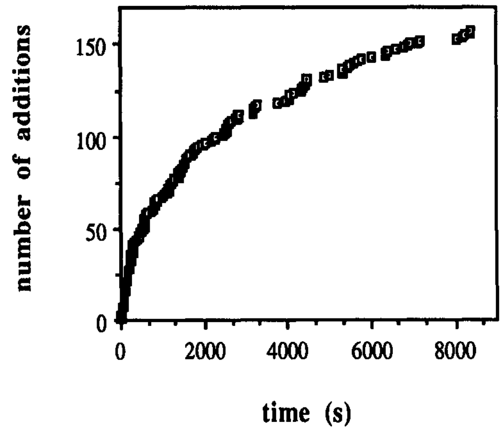

*Figure 3. Typical crystallization run obtained at controlled supersaturation using the automatic system. Constant total calcium concentration = constant total oxalate concentration = 2·7 × $10^{-4}$ M, ($NH_4Cl$) = 0·1 M, T = 37 °C, calcium stock solution concentration = oxalate stock solution concentration = 1 × $10^{-3}$ M, initial amount of calcium oxalate monohydrate seed crystals = 0·125 g/l. Each addition was 0·2 ml.*

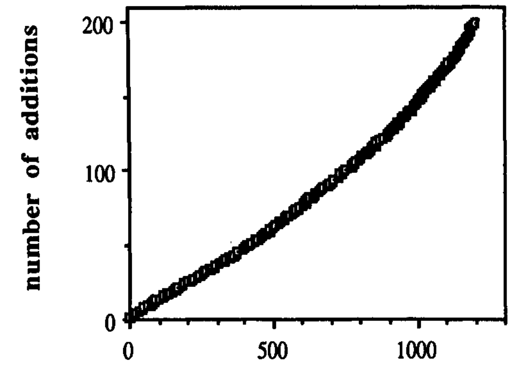

*Figure 4. Typical crystallization run obtained at controlled supersaturation using the automatic system. Constant total calcium concentration = constant total oxalate concentration = 5 × $10^{-4}$ M, ($NH_4Cl$) = 0·1 M, T = 37 °C, calcium stock solution concentration = oxalate stock solution concentration = 1 × $10^{-2}$ M, initial amount of calcium oxalate monohydrate seed crystals = 0·125 g/l. Each addition was 0·2 ml.*

Figures 5 and 6 show crystal growth rates. $R$ initially decreased (this was probably due to the blocking of some crystalline imperfections); then it becomes constant. The time interval of constant $R$ depends on the supersaturation value: the higher the supersaturation, the shorter the $R$ constant time interval. Thereafter, $R$ increased with time. $R$ values calculated by applying equations (7) and (8) had close results when moderate amounts of calcium oxalate was added. However, for the higher supersaturation experiment (see figures 4 and 6), when exceeding approximately 40 additions (which will give about 6 mg of crystallized calcium oxalate which is a 23·4% increase

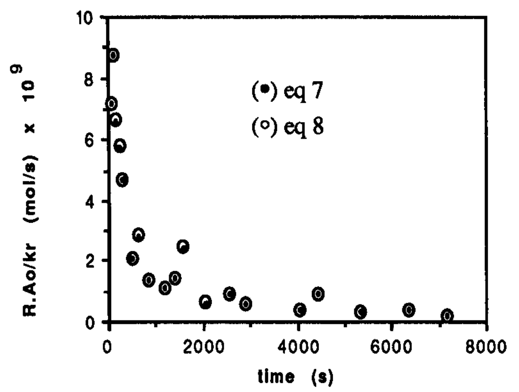

*Figure 5. Crystal growth rate under the conditions shown in figure 3. Rates were calculated using equations (7) and (8) (see text) and there was little difference between them.*

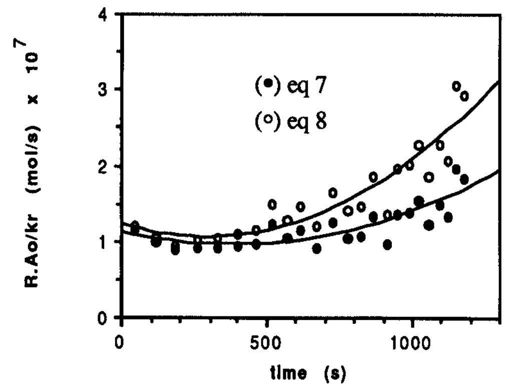

*Figure 6. Crystal growth rate under the conditions shown in figure 4. Rates were calculated using equations (7) and (8) and the results were increasingly different with time.*

in the initial weight of seed), the rate of growth per unit of crystal area was lower than the rate calculated assuming the amount of solid phase to be constant. In addition, the value of $R$ was greater when considering $R$ per unit of area. So is the increase in $R$ a consequence of the simplicity of the assumptions made to estimate crystal area increase, or does the rate of crystal growth per unit of area of crystal phase really increase with time after a relatively large amount of salt crystallization? More work on the assumptions for estimating area increase will need to be done to answer this, but it is possible that after the initial crystal growth there is a secondary nucleation crystallization mechanism.

### Conclusion

The automatic system presented in this paper permits a simple calculation of crystal growth rate and apparent order of crystallization. Preliminary results indicate that studying the kientics of crystal growth at constant supersaturation will lead to a better understanding of crystallization. The automatic crystallizer is still being developed; results from this preliminary work are sufficiently encouraging to justify more research with different substances and to work on improving the software and apparatus in order to use techniques other than potentiometry.

### Acknowledgements

This work was supported by the Direccion General de Investigacion Cientifica Y Tecnica (grant PB 89-0423).

### References

1. Addadi, L., Berkovitch-Yellin, Z., Weissbuch, I., van Mill, J., Shimon, L. J. W., Lahav, M. and Leiserowitz, L., *Angew. chem. int. Ed. Engl.*, **24** (1985), 466.
2. Addadi, L. and Weiner, S., *Molecular Crystals and Liquid Crystals*, **134** (1986), 305.
3. Nancollas, G. H., *Biological Mineralization and Demineralization* (Springer-Verlag, Berlin, 1982).
4. Nyvlt, J., Sohnel, O., Matuchova, M. and Broul, M.,in *The Kinetics of Industrial Crystallization*, Churchill, S. W. (Ed.) (Amsterdam, 1985).
5. Nancollas, G. H. and Zawacki, S. J., in *Industrial Crystallization 84*, Jancic, S. J. and de Jong, E. J. (Eds.) (Elsevier, Amsterdam, 1984).
6. Sheehan, M. E. and Nancollas, G. H., *Investigations in Urology*, **17** (1980), 446.
7. Singh, R. P., Gaur, S. S., White, D. J. and Nancollas, G. H., *J. Colloid and Interface Science*, **118** (1987), 379.
8. Singh, R. P., Gaur, S. S., Sheehan, M. E. and Nancollas, G. H., *J. Crystal Growth*, **87** (1988), 318.

